# Olive Leaf Extract (OLE) Anti-Tumor Activities Against Hematologic Tumors: Potential Therapeutic Implications for Pediatric Patients with B-Acute Lymphoblastic Leukemia

**DOI:** 10.3390/nu18010015

**Published:** 2025-12-19

**Authors:** Irma Airoldi, Lucrezia Canè, Chiara Brignole, Eleonora Ciampi, Daniela Montagna, Fabio Morandi

**Affiliations:** 1 UOSD Laboratory of Cell Therapies, IRCCS Istituto Giannina Gaslini, 16147 Genova, Italy; irmaairoldi@gaslini.org (I.A.); lucreziacane01@icloud.com (L.C.); 2 UOSD Laboratory of Experimental Therapies in Oncology, IRCCS Istituto Giannina Gaslini, 16147 Genova, Italy; chiarabrignole@gaslini.org (C.B.); eleonoraciampi@gaslini.org (E.C.); 3 Department of Sciences Clinic-Surgical, Diagnostic and Pediatric, University of Pavia, 27100 Pavia, Italy; daniela.montagna@unipv.it; 4 Pediatric Clinic, Foundation IRCCS Policlinico San Matteo, 27100 Pavia, Italy

**Keywords:** olive leaf extract, pediatric leukemias, nutraceuticals

## Abstract

**Background/Objectives:** Several studies reported that olive leaf extract (OLE) may exert potent anti-cancer activities against human solid and hematological tumors. Such effects are mostly related to the polyphenol oleuropein and its derivatives, which are highly concentrated in OLE. Here, we investigated the anti-tumor effects of OLE in vitro against human acute leukemia and lymphoma cells. **Methods:** Cell proliferation and apoptosis have been evaluated by flow cytometry (using CFSE and Annexin-V/7AAD, respectively) in the presence or absence of OLE at different concentrations and in combination with or without chemotherapeutic drugs. Cellular pathways have been analyzed using antibody arrays. **Results:** OLE inhibited cell proliferation and induced apoptosis in B-acute lymphoblastic leukemia (B-ALL) and, to a lesser extent, in lymphomas and acute myeloid leukemia (AML) cell lines. Notably, OLE-induced apoptosis also occurs in primary leukemic blasts from B-ALL patients, both at diagnosis and at relapse, but only marginally in primary AML blasts. The expression and phosphorylation of proteins involved in the induction of apoptosis were modulated by OLE in B-ALL, whereas modest effects were observed in AML. Interestingly, some proteins were modulated in opposite ways in B-ALL and AML, potentially explaining their different responses to OLE. Finally, a synergistic and additive effect was observed for OLE in combination with cytarabine, but not with cyclophosphamide. **Conclusions:** We may envisage that OLE may be used as a food supplement in B-ALL patients treated with cytarabine, taking advantage of the potentiated effect of chemotherapy, without additional side effects.

## 1. Introduction

*Olea europaea* L. (Oleaceae) is a characteristic Mediterranean species used to produce olives and oils, representing key components of the traditional Mediterranean diet that includes a high intake of fruit, vegetables, fish, red wine, and extra virgin olive oil (EVOO). Such a diet is widely recognized to have an impact on good health, long life, prevention of chronic diseases (e.g., cardiovascular and neurodegenerative), and cancers in the Mediterranean population. In addition to EVOO, another phytochemical may be obtained from the olive plant: olive leaf extract (OLE), containing similar biologically active compounds, even at much higher concentrations and a wider variety. OLE is abundant in polyphenolic compounds, broadly categorized into secoiridoids including oleuropein, dimethyloleuropein, and oleuropein–aglycone, simple phenols such as hydroxytyrosol and tyrosol, and flavonoids (e.g., apigenin and luteolin). These polyphenols have been documented to exert anti-microbial [1,2], anti-viral [3,4,5], hypoglycemic [6], neuro-protective [7,8], anti-inflammatory [9,10,11] and anti-tumor activities [12,13,14,15]. The latter function was reported both in vitro and in vivo against a wide panel of human tumors, including breast, pancreatic, colorectal, and skin cancers, as well as melanoma, glioblastoma, neuroblastoma, and acute leukemias. In the context of prostate and breast cancers, oleuropein and hydrotyrosol were the main molecules endowed with anti-tumor properties; noteworthy, it was reported that they were able to discriminate between cancer and normal cells by inhibiting proliferation and inducing apoptosis only in the former [16,17,18]. In acute leukemias, the ability of OLE to counteract leukemia cell growth was evaluated exclusively against cell lines of myeloid origin (i.e., K562 and HL-60), but not in the B-acute lymphoblastic leukemias (ALL). In addition, B-cell lymphomas have not yet been investigated. The anti-tumor activity may be exerted through different and complementary mechanisms [15] mainly related to the (i) inhibition of genotoxicity and mutagenesis, (ii) induction of apoptosis, (iii) inhibition of cell proliferation, (iv) selective cytotoxicity, (v) regulation of oxidative stress through reactive oxygen species (ROS), and (vi) inhibition of angiogenesis, tumor cell migration and metastasis. Thus, olive polyphenols can target all phases of chemoprevention from primary prevention by inhibiting cancer initiation, to secondary prevention by inhibiting cancer promotion, and therapeutic intervention by inhibiting cancer progression. The broad beneficial effects of OLE are of particular relevance in the context of oncologic pediatric patients, especially those with poor prognoses, receiving debilitating treatments that affect the patients’ quality of life.

With this background, we investigated whether OLE may function as an anti-tumor agent against pediatric B-ALL, acute myeloid leukemias (AML), and human B-cell lymphomas, investigating the cellular pathways involved. B-ALL is the most common hematological cancer in children caused by an abnormal clonal proliferation of B-cell progenitors, thus manifesting as the accumulation of malignant and poorly differentiated lymphoid cells within the bone marrow, peripheral blood, and some extra-medullary sites. Despite the optimization of treatment protocols for pediatric B-ALL, approximately 15% of patients experience a relapse, and the overall survival of these patients is less than 10%. Moreover, other patients are refractory to the treatment and do not achieve remission. Hematopoietic stem cell transplantation is almost inevitably part of salvage treatment after relapse, but transplant-related mortality and long-term morbidity remain a pitfall. The prognosis of AML, which represents the second most common hematologic malignancy in children, remains less favorable compared to ALL, and approximately 30% of AML patients experience relapse and ultimately die from disease-related complications or treatment side effects. Finally, B-cell lymphoma is one of the more common cancers among children, teens, and young adults that starts in early forms of B lymphocytes. The overall 5-year relative survival rate is approximately 74% and patients, as for leukemias, undergo intensive chemotherapy and radiotherapy, surgery, hematopoietic stem cell transplantation, and immunotherapy.

Thus, novel therapeutic approaches that are less toxic, selectively target malignant cells, and potentially strengthen the immune responses of oncologic pediatric patients affected by leukemias or lymphomas are needed.

## 2. Materials and Methods

### 2.1. OLE Composition

Aqueous OLE, whose composition has been previously reported [12], was used throughout the study and was kindly provided by Evergreen Life, San Giovanni al Natisone, Italy. The manufacturer has implemented a patented method for the extraction at very low temperatures of different active compounds, mainly polyphenols, from olive leaves. The mean phytochemical compounds and their concentrations contained in OLE were the following: Oleuropein 2656 mg/L, Hydrohytyrosol 213 mg/L, Tyrosol 174 mg/L, Elenolic Acid 1393 mg/L, and Rutin 237 mg/L. Two different batches of OLE have been used throughout this study, with minimal variations in the composition, as disclosed by the manufacturer.

### 2.2. Cell Lines and Primary Leukemias from Pediatric Patients

The following human cell lines were used: NALM-6 and 697 B-ALL; K562, THP-1, and RPMI8866 myeloid leukemias; SUDHL-4, U937, and RAJI lymphomas (ATCC and Biobank at Ospedale San Martino, Genova, Italy). Cells were cultured in RPMI-1640 (Gibco/Thermo Fisher Scientific, Waltham, MA, USA) supplemented with 10% of heat-inactivated fetal bovine serum (FBS, by Euroclone, Milano, Italy), 50 IU/mL penicillin, 50 g/mL streptomycin sulphate, and 2 mM L-glutamine (Euroclone, Milano, Italy). Fresh medium was replaced every three days.

Leukemia cells (five B-ALL and three AML) were derived from patients’ heparinized bone marrow aspirates (>90% blast cells) at diagnosis and, in patients experiencing disease recurrence, also at relapse, following written informed consent according to Helsinki Declaration. Leukemia cells were isolated by Ficoll-Hypaque Lympholyte^®^ (Cedarlane, Burlington, ON, Canada) density gradient centrifugation, then cryopreserved in FBS (Euroclone SPA, Milan, Italy), with 10% DMSO (AL.CHI.MI.A S.R.L., Padua, Italy) for later use. Characteristics of patients are summarized in Table 1.

### 2.3. Cell Proliferation

Since oleuropein was the most effective and the most concentrated polyphenol in OLE, the concentration of OLE reported throughout the study was referred to the final concentration of oleuropein. Leukemia and lymphoma cell proliferation was assessed by flow cytometry using a CellTrace™ CFSE Cell Proliferation Kit (Thermo Fisher Scientific, Waltham, MA, USA). Briefly, cell lines were resuspended in RPMI-1640 without serum, stained with Carboxyfluorescein diacetate *N*-succinimidyl ester (CFSE) at a final concentration of 2 μM, and incubated at 37 °C for 15 min. After washing in RPMI 10% FBS, cells were resuspended in RPMI 10% FBS, seeded in a 96-well plate (10^5^/well), and treated with OLE at the final concentrations of 50, 100, 200, and 400 µM. After 6 days of culture, cells were washed and analyzed for cell proliferation, as witnessed by CFSE dilution, by flow cytometry. Cells cultured for 6 days in the absence of FBS were used as negative controls. Cells were harvested, washed, and resuspended in 300 μL of MACS buffer (PBS supplemented with 2 μM EDTA and 0.5% BSA). Samples were then run on a Gallios^®^ flow cytometer (Beckman Coulter, Brea, CA, USA), acquiring at least 3 × 10^4^ events, and data were analyzed using Kaluza^®^ analysis software version 2.0 (Beckman Coulter, Brea, CA, USA). The results were expressed as CFSE mean fluorescence intensity (MFI).

### 2.4. Leukemia and Lymphoma Cell Apoptosis

Leukemia and lymphoma cell lines, as well as primary samples from pediatric leukemia patients, were analyzed for induction of apoptosis driven by OLE. Cells were seeded at 105/well in 96-well plates, treated for 24, 48, and 72 hours (h) with OLE 50, 100, 200, and 400 µM, and tested for apoptosis by flow cytometry using the Annexin V-FITC/7AAD kit (Beckman Coulter, Brea, CA, USA), following the manufacturer’s instructions. Briefly, 10^5^ cells were washed with PBS and resuspended in 100 μL of binding buffer 1X. Next, 7-AAD and FITC-conjugated Annexin V were added, and cells were incubated for 15 min on ice. Next, 300 μL of binding buffer 1X was added to each sample, and cells were run on a Gallios^®^ flow cytometer (Beckman Coulter, Brea, CA, USA), acquiring at least 3 × 10^4^ events. Data were analyzed using Kaluza^®^ analysis software (Beckman Coulter, Brea, CA, USA). Annexin V^+^ staining identified early apoptotic cells, 7AAD^+^/Annexin^+^ the late apoptotic cells, and 7AAD^+^/Annexin^−^ dead cells. B-ALL and AML primary samples were treated for 24 h with 100 and 200 µM OLE and tested for apoptosis.

### 2.5. OLE Molecular Pathways in Leukemia Cells

To investigate the molecular pathways involved in the apoptosis induced by OLE in leukemia cells, we selected the NALM-6 cells, due to their high response to OLE in terms of inhibition of cell proliferation and induction of apoptosis, and the K562 cell line for its low response to OLE treatment. Cells were treated in the presence or absence of 200 µM OLE for 24 h, and protein extracts were obtained following the protocol reported in each human proteome profiler kit. Proteins were quantified using the Bio-Rad Protein Assay Dye Reagent (Bio-Rad, Hercules, CA, USA) and Tecan Infinite 200 Pro Bioreader (Tecan, Männedorf, Switzerland) (absorbance at 555 nm). A total mass of 200 µg of each protein lysate were assessed on 4 different human proteome profiler kits following the manufacturer’s instructions (Bio-Techne, Minneapolis, MN, USA): (i) apoptosis array detecting the relative levels of expression of 35 apoptosis-related proteins, (ii) NFkB pathway array analyzing 41 different proteins and 4 serine or tyrosine phosphorylation sites antibodies, (iii) phospho-kinase array with 37 different kinases and 2 related total proteins, and (iv) cell stress array analyzing 26 different stress-related proteins. The relative amount of each individual protein (corresponding to each spot tested in duplicate) was calculated using Image Lab software (Bio-Rad, version 6), upon normalization to internal controls. The fold increase was calculated for each protein as the ratio between values obtained in treated versus untreated cells. A fold increase of 1 indicated no modulation, whereas positive values indicated an up-regulation and negative values indicated a down-regulation. An arbitrary cut-off value for fold increase (1.8) was established, and only proteins with a fold increase >1.8 or >−1.8 are shown.

### 2.6. Combined Effect of OLE and Chemotherapeutic Agents on B-ALL Cell Lines

NALM-6 and 697 B-ALL cell lines were treated for 24 and 48 h with a combination of 50 or 100 µM OLE and cytarabine (Accord, 0.5 and 1 µM) or cyclophosphamide (Endoxan, Baxter, 0.5 and 1 µg/mL), obtained as a spare aliquot after therapeutic use. The percentage of live cells (7AAD^−^/Annexin V^−^) was analyzed, as described above. The combined effect of OLE with the other drugs was analyzed using ZIP and Bliss models for additive effects and HSA and Loewe models for synergistic effects. These analyses were carried out using the SynergyFinder+ web application (https://synergyfinder.org) [19,20,21,22,23].

### 2.7. Statistical Analysis

Statistical analysis was carried out using Prism 5.03 software (GraphPad Inc., Boston, MA, USA). Data distribution was analyzed using the KS normality test and the D’Agostino and Pearson omnibus normality test. Comparison of data sets was analyzed using a *t*-test or Mann–Whitney test, depending on data distribution. *p*-values < 0.05 were considered statistically significant.

## 3. Results

### 3.1. OLE Inhibits the Proliferation of Leukemia/Lymphoma Cell Lines

**Figure 1:**
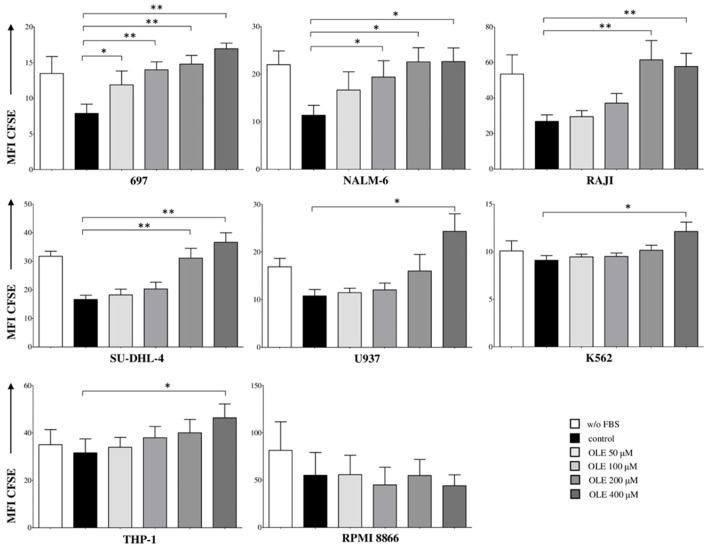
Anti-proliferative effects of OLE on leukemia and lymphoma cell lines. Different cell lines, namely 697 and NALM-6 (B-ALL), RAJI, SUDHL-4, and U937 (lymphomas), K562, THP-1 and RPMI8866 (myeloid leukemias) were stained with CFSE and cultured for 6 days in RPMI without FBS (white bars) or supplemented with 10% FBS (black bars) in the presence or absence of OLE at different concentrations (50, 100, 200 and 400 μM, grey bars). Cell proliferation was assessed by flow cytometry as CFSE dilution. Results are indicated as MFI for CFSE. Mean of six different experiments + SE is shown. Asterisks indicate a statistically significant difference (* *p* ≤ 0.05; ** *p* ≤ 0.01).

First, we tested whether OLE could affect the proliferation of leukemia/lymphoma cells. To this end, we analyzed a panel of cell lines, including B-ALL (697 and NALM-6), Burkitt’s lymphoma (RAJI), myeloid leukemia (K562, RPMI8866, and THP-1), and histiocytic lymphoma (SU-DHL-4 and U937), treated for 48 h with different concentrations of OLE. As shown in Figure 1, the proliferation of B-ALL cell lines was significantly inhibited by OLE at different concentrations, as witnessed by the increase in MFI CFSE. In detail, the proliferation of the 697 cell line (CFSE MFI mean ± SE 7.88 ± 1.29) was significantly reduced by OLE at 50 μM (11.87 ± 1.95, *p* = 0.03), 100 μM (13.98 ± 1.12, *p* = 0.0076), 200 μM (14.79 ± 1.22, *p* = 0.0043), and 400 μM (16.94 ± 0.79, *p* = 0.0011). Similarly, the proliferation of the NALM-6 cell line (11.36 ± 2.08) was significantly inhibited by OLE at 100 μM (19.38 ± 3.44, *p* = 0.01), 200 μM (22.58 ± 2.97, *p* = 0.01), and 400 μM (22.64 ± 2.87, *p* = 0.0011), but not at 50 μM (16.67 ± 3.82).

The effect of OLE on the proliferation of other cell lines was more limited at the same time point and concentrations. In particular, RAJI cell line proliferation (26.84 ± 3.67) was found to be significantly reduced by OLE only at 200 μM (61.46 ± 10.9, *p* = 0.0079) and 400 μM (57.74 ± 7.4, *p* = 0.004). Other OLE concentrations proved less effective. Similar results were obtained in histiocytic lymphoma cell lines SU-DHL-4 (CTR 16.63 ± 1.47; OLE 200 μM 31.1 ± 3.41, *p* = 0.0076; OLE 400 μM 36.62 ± 3.35, *p* = 0.0011), and U937 (CTR 10.78 ± 1.34; OLE 400 μM 24.32 ± 3.69, *p* = 0.01) cell lines.

Myeloid leukemia cells lines resulted the most resistant to OLE treatment, in particular, proliferation of K562 and THP-1 cell lines was inhibited only at the highest dose of OLE (K562: CTR 9.1 ± 0.5; OLE 400 μM 12.13 ± 0.98, *p* = 0.01; THP-1: CTR 31.59 ± 5.91; OLE 400 μM 46.38 ± 5.82, *p* = 0.04), whereas no modulation of cell proliferation was observed in RPMI8866 cell at any OLE concentration tested.

### 3.2. OLE-Induced Apoptosis in Leukemia/Lymphoma Cell Lines

**Figure 2:**
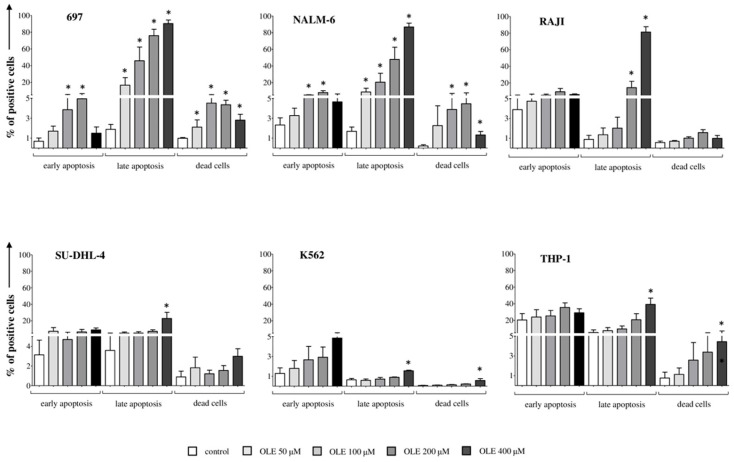
Pro-apoptotic effects of OLE on leukemia and lymphoma cell lines. The same leukemia/lymphoma cell lines tested in proliferation assay were analyzed for apoptosis upon treatment with OLE. Cells were cultured for 48 h in RPMI supplemented with 10% FBS (white bars) in the presence or absence of OLE at different concentrations (50, 100, 200 and 400 μM, grey bars). Apoptosis was assessed by staining with 7AAD and Annexin-V FITC and flow cytometric analysis. Results are indicated as % of Annexin V^+^ cells (early apoptosis), Annexin V^+^/7AAD^+^ cells (late apoptosis) and 7AAD^+^ cells (dead cells). Mean of six different experiments + SE is shown. Asterisks indicate a statistically significant difference (* *p* ≤ 0.05).

Next, we investigated the ability of OLE to induce apoptosis of leukemia/lymphoma cells, at the same concentrations used for proliferation studies, after 24, 48, and 72 h of treatment. OLE-induced cell apoptosis at any time point tested, and data from 48 h treatment (i.e., intermediate time) are reported in Figure 2. On the basis of the results obtained on cell proliferation, we first analyzed the B-ALL cell lines. The percentage of 697 cells in early apoptosis after 48 h of culture (% of Annexin V^+^/7AAD^−^ cells, mean ± SE 0.68 ± 0.32) was significantly increased by OLE at 100 μM (3.86 ± 1.75, *p* = 0.05) and 200 μM (4.95 ± 1.66, *p* = 0.05). In addition, the percentage of 697 cells in late apoptosis (Annexin V^+^/7AAD^+^ cells, 1.88 ± 0.49) was significantly increased in the presence of OLE at the concentration of 50 μM (16.72 ± 9, *p* = 0.05), 100 μM (45.88 ± 16.44, *p* = 0.05), 200 μM (75.97 ± 7.61, *p* = 0.05), and 400 μM (90.36 ± 4.28, *p* = 0.05). The percentage of dead cells (Annexin V^−^/7AAD^+^ cells, 0.98 ± 0.07) was also significantly increased by OLE at 50 μM (2.1 ± 0.73, *p* = 0.05), 100 μM (4.52 ± 0.89, *p* = 0.05), 200 μM (4.36 ± 0.44, *p* = 0.05), and 400 μM (2.8 ± 0.59, *p* = 0.05). Similar results were obtained by analyzing the NALM-6 cell line, where the percentage of cells in early apoptosis after 48 h of culture (2.32 ± 0.72) was significantly increased by OLE at 100 μM (5.1 ± 0.45, *p* = 0.05) and 200 μM (8.12 ± 2.15, *p* = 0.05). In addition, the percentage of cells in late apoptosis (1.69 ± 0.43) was significantly increased by OLE at 50 μM (8.79 ± 4.52, *p* = 0.05), 100 μM (20.6 ± 10.6, *p* = 0.05), 200 μM (47.99 ± 14.51, *p* = 0.05), and 400 μM (86.83 ± 4.66, *p* = 0.05), as well as of dead cells (0.21 ± 0.12) that was significantly increased by OLE at 100 μM (3.9 ± 3.14, *p* = 0.05), 200 μM (4.47 ± 3.2, *p* = 0.05), and 400 μM (1.32 ± 0.35, *p* = 0.05).

Similarly to that reported in cell proliferation studies, limited effects were observed in terms of induction of apoptosis in RAJI and SU-DHL-4 lymphoma cell lines, where we observed only an increased percentage of cells in late apoptosis (0.88 ± 0.4 and 3.59 ± 1.62, respectively). Such an increase was observed in RAJI cells in the presence of OLE at 200 μM (14.32 ± 7.62, *p* = 0.05) and 400 μM (81.31 ± 6.45, *p* = 0.05) and in the SU-DHL-4 cell line at 400 μM OLE (22.94 ± 7.3, *p* = 0.05). We obtained similar results also in RPMI8866 and U937 cells.

Finally, we analyzed the myeloid leukemia cell lines. As expected, we did not observe any induction of early apoptosis in the K562 cell line, but only a slight increase of cells in late apoptosis (0.64 ± 0.13) and of dead cells (0.07 ± 0.01) in the presence of 400 μM OLE (1.55 ± 0.06 and 0.58 ± 0.18, respectively, *p* = 0.05). Superimposable results were obtained by analyzing the THP-1 cell line, with an increase in cells in late apoptosis (5.32 ± 3.16) and dead cells (0.76 ± 1.95) with 400 μM OLE (39.23 ± 7.53 and 4.44 ± 2.78, respectively, *p* = 0.05).

### 3.3. OLE-Induced Apoptosis in Primary Blasts from Leukemia Patients

**Figure 3:**
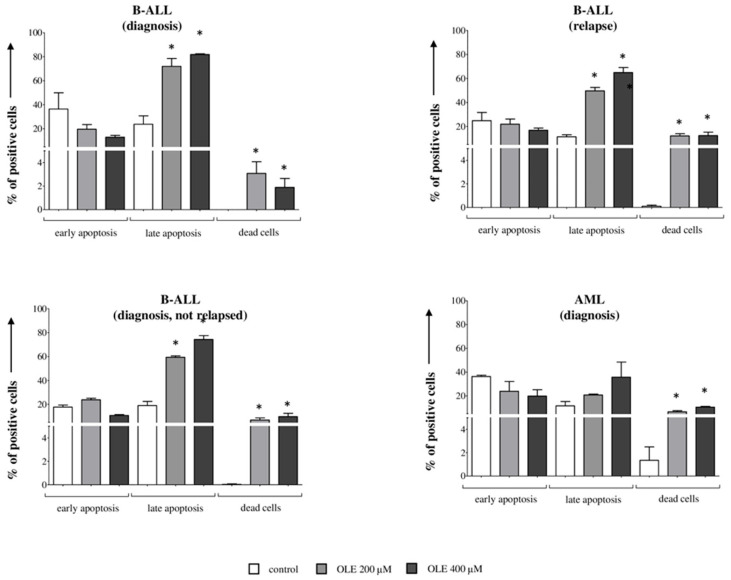
Pro-apoptotic effects of OLE on primary leukemia cells. Leukemic blasts from B-ALL patients at onset, relapse, onset with good prognosis (retrospectively not relapsed) and from AML patients at onset were cultured for 24 h in RPMI supplemented with 10% FBS (white bars) in the presence or absence of OLE at different concentrations (200 and 400 μM, grey and black bars, respectively). Apoptosis was assessed by flow cytometry by staining with 7AAD and Annexin-V FITC. Results are indicated as % of Annexin V^+^ cells (early apoptosis), Annexin V^+^/7AAD^+^ cells (late apoptosis) and 7AAD^+^ cells (dead cells). Mean of experiments performed on three different patients for each group + SE is shown. Asterisks indicated a statistically significant difference (* *p* ≤ 0.05).

To validate the results obtained with cell lines, we analyzed apoptosis of leukemic blasts obtained from patients with B-ALL and AML after 24 h of culture, in the presence or absence of OLE (200 and 400 μM). As shown in Figure 3, the percentage of leukemic blasts from patients with B-ALL at diagnosis in late apoptosis (% of Annexin V^+^/7AAD^−^ cells, mean ± SE 23.81 ± 6.99) was significantly increased by OLE at 200 μM (72 ± 6.53, *p* = 0.05) and 400 μM (81.91 ± 0.56, *p* = 0.05). The percentage of dead cells (% 7AAD^+^ cells, mean ± SE 0.1 ± 0.1) was also significantly increased by OLE at 200 μM (3.08 ± 0.98, *p* = 0.05) and 400 μM (1.89 ± 0.75, *p* = 0.05). We obtained superimposable results from the same B-ALL patients at relapse, with a significantly higher percentage of both apoptotic cells (11.47 ± 1.69) and dead cells (0 ± 0.1) in the presence of OLE at 200 μM (49.74 ± 2.84, *p* = 0.05 and 12.2 ± 1.8, *p* = 0.05, respectively) and 400 μM (64.94 ± 4.14, *p* = 0.05 and 12.47 ± 2.87, *p* = 0.05, respectively). Regarding the leukemic blasts from patients who maintained disease remission, the percentage of blasts in late apoptosis (19.03 ± 3.42) was significantly increased by OLE at 200 μM (59.38 ± 1.2, *p* = 0.05) and 400 μM (74.3 ± 3.2, *p* = 0.05), as well as the percentage of dead cells (ctr: 0.04 ± 0.04; OLE 200 μM: 6.9 ± 1.7, *p* = 0.05; OLE 400 μM: 9.74 ± 2.74, *p* = 0.05).

Next, we analyzed leukemic blasts from patients with AML. In contrast to ALL, but consistent with that reported for AML cell lines, OLE did not induce a significant increase in apoptotic cells, but only a slight increase in dead cells (1.35 ± 1.15) either at 200 μM (6.7 ± 0.9, *p* = 0.05) and 400 μM (10.6 ± 0.62, *p* = 0.05).

These results were in accordance with those obtained with B-ALL and AML cell lines and confirm that B-ALL is the most sensitive acute leukemia to OLE treatment.

### 3.4. OLE Differentially Modulated Cellular Pathways in B-ALL and AML Cell Lines

To determine which cellular pathways may be affected in response to OLE treatment, we performed antibody array analyses on cellular lysates from the NALM-6 and K562 cell lines, the most treatment-sensitive and resistant, respectively. To this end, cells were cultured in the presence of OLE 200 μM for 24 h. The arrays selected included 139 molecules involved in cellular stress and apoptosis.

**Figure 4:**
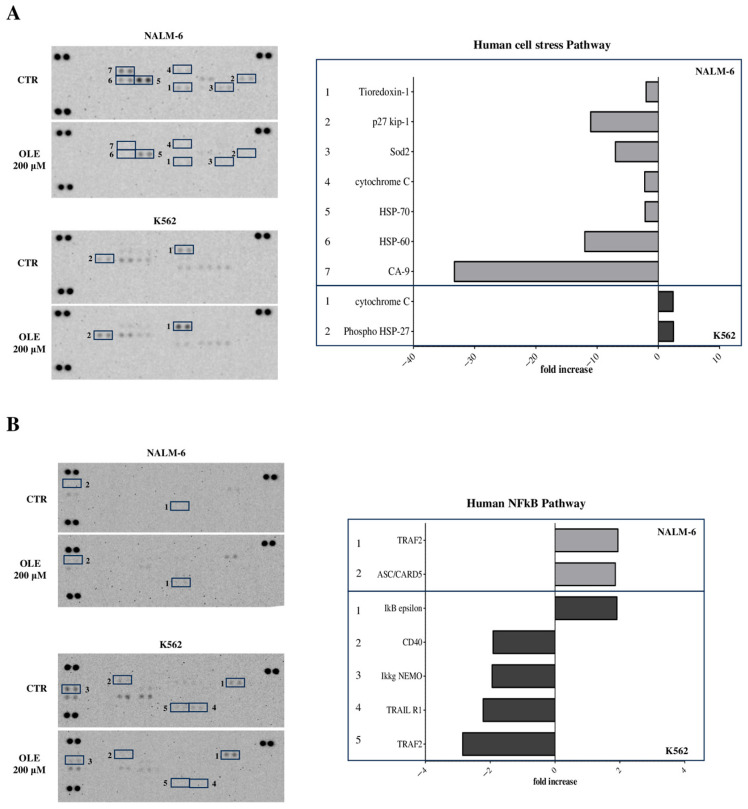
Modulation of molecules involved in the cellular stress pathways by OLE in B-ALL and AML cell lines. Antibody array was performed to analyze cellular stress (**A**) and NFkB (**B**) pathways on cellular lysates from NALM-6 and K562 cell lines cultured for 24 h in RPMI supplemented with 10% FBS in the presence or absence of OLE at 200 μM. Raw blots are shown in the left panels, where spots for each protein (in duplicate) are numerated. On the right panels fold increase (>1.8) and decrease (<−1.8) are shown for each individual protein, as numerated in the left panels. Grey bars indicated proteins modulated in NALM-6 cell line, whereas black bars indicated proteins modulated in K562 cell line.

First, we investigated the expression of different proteins involved in cellular stress. As shown in Figure 4A, the expression of different proteins was down-modulated in the NALM-6 cell line after OLE treatment, including Carbonic Anhydrase (CA-9, −33.3), Heat Shock Protein (HSP)-60 (−12.2), p27/kip-1 (−11.06), Superoxide Dismutase (SOD)2 (−7.01), cytochrome C (−2.23), HSP-70 (−2.17), and Tioredoxin-1 (−2.01). On the contrary, a limited effect of OLE was observed in K562 cells, with a moderate increase of cytochrome C (fold increase: 2.41) and phospho-HSP-27 (2.46) proteins.

The analysis of the NFkB pathway revealed that OLE-induced up-regulation of a few molecules, including TRAF2 (1.94) and Apoptosis-associated Speck-like protein containing CARD (ASC/CARD5, 1.86) in the NALM-6 cell line (Figure 4B), whereas a higher number of molecules were modulated by OLE in K562 cells. In particular, the expression of IkB epsilon was increased (1.91), and that of Tumor Necrosis Factor Receptor Associated Factors (TRAF)2 (−2.85), TNF-Related Apoptosis-Inducing Ligand Receptor (TRAIL R)1 (−2.22), CD40 (−1.91), and Ikkg/NEMO (−1.94) was down-regulated.

**Figure 5:**
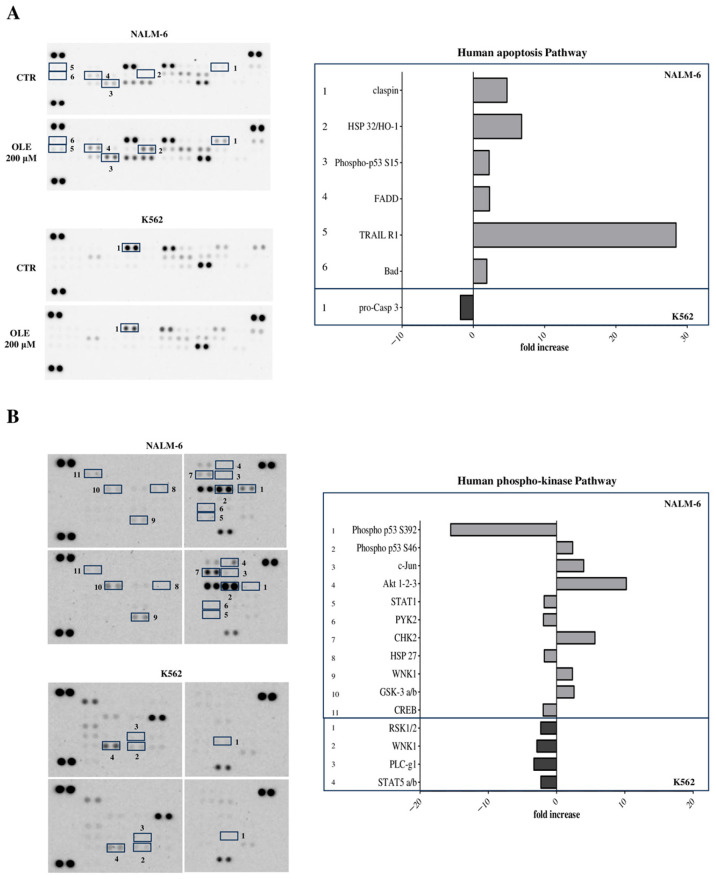
Impact of OLE on apoptosis pathways and phosphorylation of kinases in B-ALL and AML cell lines. Antibody array was performed to analyze apoptosis (**A**) and phosphokinase (**B**) pathways on cellular lysates from NALM -6 and K562 cells cultured for 24 h in RPMI supplemented with 10% FBS in the presence or absence of OLE at 200 μM. Raw blots are shown in the left panels, where spots for each protein (in duplicate) are numerated. On the right panels fold increase (>1.8) and decrease (<−1.8) are shown for each individual protein, as numerated in the left panels. Grey bars indicated proteins modulated in NALM-6 cell line, whereas black bars indicated proteins modulated in K562 cell line.

Next, we analyzed the apoptotic pathway. As shown in Figure 5A, the expression of multiple proteins was up-regulated by OLE in NALM-6 cells, including TRAIL R1 (28.43), Heme Oxygenases (HO)-1/HSP-32 (6.77), clapsin (4.72), phospho-p53 S15 (2.22), Fas Associated via Death Domain (FADD, 2.27), and Bad (1.89), whereas in K562 cell line we observed only a slight down-modulation of pro-caspase 3 (−1.81). Finally, we analyzed the phospho-kinase pathway and, again, the expression of different protein was affected by OLE in NALM-6, with an increased phosphorylation of Akt 1-2-3 (10.22), CHK-2 (5.62), WNK1 (2.32), Glycogen Synthase Kinase (GSK)-3 α/β (2.55), c-jun (3.97), and phospho-p53 s46 (2.36) and decreased phosphorylation of phospho-p53 s392 (−15.51), STAT1 (−1.82), PYK2 (−1.92), HSP-27 (−1.8), and cAMP Response Element-binding protein (CREB, 1.97). In contrast, the phosphorylation of only a few kinases was decreased by OLE in K562 cells, including RSK1/2 (−2.33), WNK1 (−2.88), PLC-γ1 (−3.32), and STAT5 α/β (−2.3).

### 3.5. Induction of Apoptosis in B-ALL Cells by OLE in Combination with Chemotherapeutic Drugs

On the basis of previous results, we focused on B-ALL cells to assess the potential additive and/or synergistic pro-apoptotic activity of OLE in combination with chemotherapeutic drugs commonly used to treat leukemia patients. To this end, we selected cytarabine and cyclophosphamide/endoxan as chemotherapeutic agents that, in our in vitro experiments, were used at concentrations of 0.5 and 1 μM and of 0.5 and 1 μg/mL, respectively. At the same time, NALM-6 and 697 cells were treated alone or in combination with OLE at 50 or 100 μM. The percentage of 7AAD-/Annexin V-alive cells was evaluated by flow cytometry for each experimental condition, and data were analyzed using the SynergyFinder+ web application. The additive and synergistic effects were analyzed by the ZIP and HSA models, respectively.

**Figure 6:**
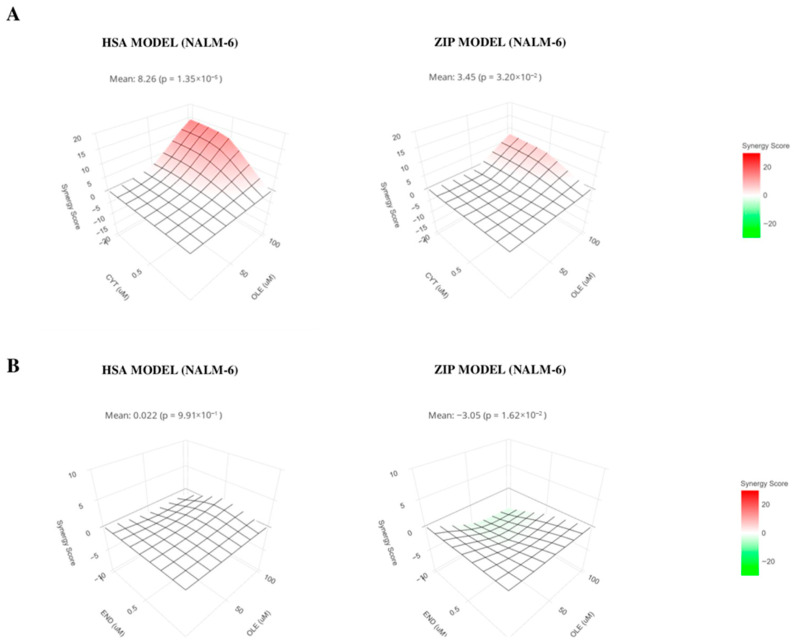
OLE effects on B-ALL in combination with chemotherapeutic drugs. NALM-6 cell line was cultured 48 h in the presence or absence of OLE (50 and 100 μM), cytarabine (cyt 0.5 and 1 μM) and cyclophosphamide/endoxan (end 0.5 and 1 μg/mL) and the percentage of alive cells was evaluated for each experimental combination. Synergistic effects (left panels) were analyzed by HSA model for OLE/cytarabine (**A**) and OLE/end (**B**). Additive effects (right panels) were analyzed using the ZIP model for OLE/cytarabine (**A**) and OLE/end (**B**). Means and *p* values are shown.

As shown in Figure 6A, the HSA model highlighted a synergistic effect of OLE in combination with cytarabine on NALM-6 cells (left panel, mean score: 8.26, *p* = 1.35 × 10^−5^). A significant additive effect was also observed using the ZIP model (right panel, score: 3.45, *p* = 0.03). Conversely, experiments performed with OLE and cyclophosphamide/endoxan revealed no synergistic effects (Figure 6B) using the HSA model (left panel, score 0.022, *p* = 0.99), whereas the ZIP model suggested an antagonistic activity (right panel, score −3.05, *p* = 0.016).

**Figure 7:**
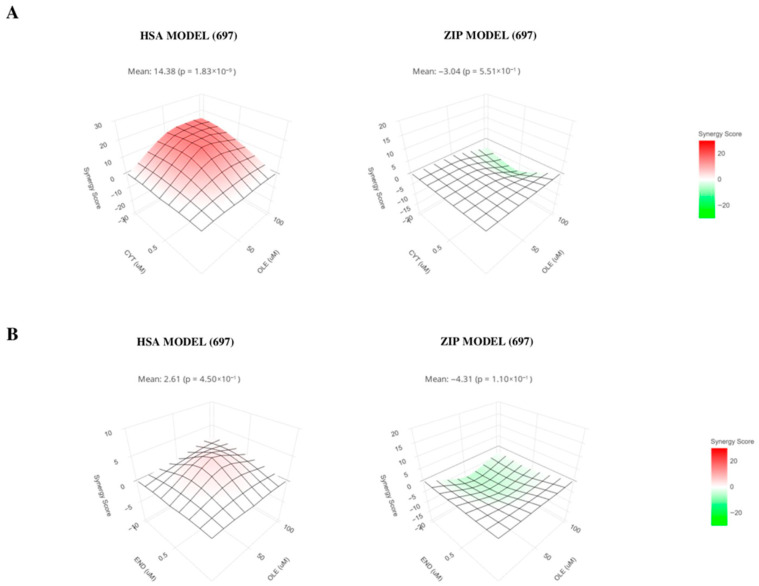
OLE anti-tumor activity against AML in combination with chemotherapeutic drugs. 697 cell line was cultured 48 h in the presence or absence of OLE (50 and 100 μM) in combination with cytarabine (cyt 0.5 and 1 μM) or cyclophosphamide/endoxan (end 0.5 and 1 μg/mL) and the percentage of alive cells was evaluated for each experimental combination. Synergistic effects (left panels) were analyzed by HSA model for OLE/cytarabine (**A**) and OLE/end (**B**). Additive effects (right panels) were analyzed using the ZIP model for OLE/cytarabine (**A**) and OLE/end (**B**). Means and *p* values are shown.

As shown in Figure 7A, similar results were obtained with the 697 cells, since a synergic effect emerged with a combined treatment of OLE and cytarabine (left panel, HSA model mean score: 14.38, *p* = 1.83 × 10^−9^) and no significant additive activity (right panel, ZIP model, score: −3.04, *p* = 0.55). No synergistic or additive effect emerged from the combined treatment with OLE and cyclophosphamide/endoxan (Figure 7B; HSA model score 2.61, *p* = 0.45 on the left; ZIP model score −4.31, *p* = 0.11).

Similar results were obtained using Bliss and Loewe models to analyze synergistic or additive effects, respectively (Supplementary Figures S1 and S2), as shown in the heat maps reported in Supplementary Figures S3–S6.

## 4. Discussion

OLE has attracted much interest in recent years, since different studies demonstrated its efficacy in anti-cancer treatment [12,24,25,26,27,28,29,30,31]. Indeed, OLE contains a high concentration of several molecules with a direct activity against solid and hematological tumors, and/or indirect anti-tumor effects via an increase in chemotherapy efficacy, such as oleuropein [32,33,34,35,36,37,38,39,40,41,42], rutin [43,44,45,46,47,48,49], hydrohytyrosol [50,51,52,53,54], and tyrosol [55,56]. Among them, oleuropein represents the main polyphenol and the most effective molecule against tumor cells [31]. The concentration of these compounds in OLE used in this study is reported in Section 2, and the variability of these concentrations is very low among different batches. However, the standardization of the extract is crucial for future preclinical and clinical studies. So far, only a few studies have investigated the effects of OLE or oleuropein in the context of human leukemias [57,58,59,60,61]. Here, we reported that OLE has anti-proliferative and pro-apoptotic effects in vitro on B-ALL cell lines, whereas such activities were limited in AML and lymphoma cell lines. Furthermore, the induction of apoptosis and cell death in both B-ALL and AML cell lines was dose-dependent. More importantly, similar pro-apoptotic effects were also observed in primary leukemic blasts from patients with B-ALL but not in those from AML patients.

To clarify the molecular mechanisms underlying the anti-proliferative and pro-apoptotic effects of OLE against acute leukemias, we investigated the involvement of different cellular pathways using a panel of antibody arrays assessing the modulation of 102 molecules involved in the cellular stress, NFkB pathway, and apoptosis, as well as 37 phosphorylated kinases. In all of these pathways, we found a different OLE-mediated modulation between B-ALL and AML cell lines, which may explain the different and selective effects observed on proliferation and apoptosis. Indeed, B-ALL cells, more than AML cells, responded to OLE and showed greater inhibition of cell proliferation and induction of apoptosis. Such biological effects were paralleled by an increase in 6/35 and a decrease in 1/35 molecules involved in apoptotic processes in the B-ALL NALM-6 and in the AML K562 cells, respectively. In particular, the strongest effect observed in NALM-6 may be ascribed to the reduction of proteins known to be associated with the inhibition of proliferation and induction of apoptosis of cancer cells [62,63]. CA-9 is one of the most important proteins promoting different phases of cancer development, which is overexpressed in response to tumor hypoxia in many cancers. It plays a critical role in hypoxia-associated tumor acidosis and development of the metastatic phenotype, thus representing an attractive and promising target for systemic anticancer therapy [64]. Interestingly, it has recently been reported that a close relationship exists between CA-9, GSK3β, and AKT, which we found all modulated in the B-ALL NALM-6 cells [65]. The authors investigated the mechanism of action of ibuprofen on hypoxic cancer cells and whether ibuprofen may influence CA-9. They demonstrated that the drug down-regulated CA-9 and that such an effect was a consequence of the increased phosphorylation of AKT that led to increased inhibitory phosphorylation of GSK3β at Serine 9 (pS9-GSK3β) [65]. Since these modulations have also been observed in our study, we may speculate that the same pathway occurred in the induction of apoptosis and cell death of B-ALL cells upon treatment with OLE. Nonetheless, it is conceivable that additional molecules participated in the final anti-tumor effects driven by OLE. Indeed, four additional proteins related to cellular stress were down-regulated by OLE, namely HSP-60 and -70, SOD2, and Tioredoxin-1. HSP-60 and -70 are abundantly present in cancer, providing malignant cells a selective advantage through the suppression of apoptotic pathways, by regulating necrosis, interfering with tumor immunity, promoting angiogenesis, and supporting metastasis [66,67]. HSP-60 inhibits apoptosis mainly through a mitochondrial signal peptide [68], whereas HSP-70 prevents the activation of caspase [69]. Furthermore, the loss of these HSPs in normal cells, or their down-modulation in tumor cells, increases p53 expression and activates p53-dependent apoptosis, and finally, cell death [67,70]. Notably, in our study, we found that OLE modulated the phosphorylation of p53 (see below).

SOD2 is a member of the SOD family of antioxidants, which play an essential role in cellular protection against mitochondrial oxidative damage [71]; thus, its inhibition may impair the mitochondrial functions [72], increasing ROS and finally inducing apoptosis. In this context, HO-1/HSP32 and Bad, two molecules associated with oxidative stress and consequent apoptosis [73], were up-regulated by OLE, while not increasing the amount of cytochrome C. However, it is worth mentioning that we assessed the modulation of all these molecules in total cellular extracts; thus, we cannot exclude that the subcellular compartmentalization of cytochrome C changed upon OLE treatment and, in particular, its release from mitochondria. Tioredoxin-1 physiologically inhibits apoptosis by regulating the activities of caspases and procaspases through S-nitrosylation, and its inhibition was associated with the induction of apoptosis of tumor cells [74]. Controversial effects may be ascribed to p27/Kip1, down-modulated by OLE in NALM-6 cells, which was identified as a pivotal player during metabolic stress. Accordingly, p27 is known to be involved in pathways correlated with autophagy and apoptosis [75], although with dual effects depending on the cellular context. Notably, Russo et al. [76] investigated the mechanisms by which polyphenols interact with cellular processes by modulating the signal transduction pathways. They reported that the majority of polyphenols induced a p27 increase in a panel of tumor cell lines (e.g., promyelocytic leukemia, ovarian carcinoma, and multiple myeloma), but not in pulmonary mesothelioma and breast carcinoma. Nonetheless, the final result was the induction of apoptosis and cell cycle arrest in all cell lines.

Six out of the 35 proteins analyzed and associated with the induction of apoptosis have been found to be up-regulated in B-ALL upon OLE treatment, whereas only one was modulated in K562 cells. Among them, a strong up-regulation of TRAIL R1 was paralleled by the increase of FADD and TRAF2. The apoptosis induced by the TRAIL-R1 pathway needs the recruitment of the adaptor protein FADD to the cytoplasmic region of the receptor, followed by recruitment of pro-caspase-8 or pro-caspase-10. The formation of this death-inducing signaling complex triggers cleavage and activation of caspase-8 or caspase-10, which in turn activate downstream caspase-3, -6, and -7, and apoptosis [77]. Although we have not studied the modulation of caspases in the present work, we may hypothesize that this apoptotic pathway may also be utilized by OLE. In addition, the observed up-regulation of TRAIL R1 may render B cells sensitive to TRAIL-induced apoptosis [78].

The NFkB pathway was studied since we have previously reported that OLE-induced apoptosis in neuroblastoma cell lines by activating NFkB through phosphorylation of the p65 subunit [12]. In B-ALL cells, we did not observe this modulation, whereas we observed an increase in TRAF2 and ASC/CARD5. TRAF2, which is a key player in cancer cell survival, has been described as a tumor promoter, but may also act as a tumor suppressor [79]. Thus, it is conceivable that the regulation of TRAF2 induced by OLE may be implicated in the inhibition of proliferation and induction of apoptosis in our experimental setting. ASC/CARD5, which is an adaptor protein involved in the activation of apoptosis, was also up-regulated, as well as FADD, another crucial adaptor protein connecting death receptors to caspases [80]. Notably, upon OLE treatment of the K562 AML cell line, we highlighted a negative modulation of five different molecules, all involved in the TRAF2 signaling (i.e., TRAF2, TRAIL R1, IkB epsilon, CD40, and Ikkg/NEMO) [81,82]. These data are consistent with the opposite effects driven by OLE on B-ALL and AML cells.

Regarding the phosphorylation pattern, we detected a peculiar modulation of p53, a key regulator of apoptosis, affected by OLE in B-ALL, with enhanced phosphorylation at S15 and S46 and decreased phosphorylation at S392. It has been reported that the phosphorylation of the S46 (and also S15) residue is crucial for p53 activation and its binding to DNA and transcription of apoptotic target genes [83,84,85]. In contrast, phosphorylation of S392 is required for the mitochondrial localization and transcription-independent apoptotic function of p53 [86]. Thus, the down-modulation of the p53 phosphorylation at this site suggested that in our experimental setting, p53 predominantly acts in the nucleus through the activation of transcription of apoptosis-related genes. In addition, OLE induced the phosphorylation (and thus activation) of CHK2, which is a tumor suppressor involved in the induction of apoptosis [87]. Two additional factors that may lead either to apoptosis or survival of cancer cells have been found to increase, and are STAT1 [88] and WNK1 [89]. Conversely, the phosphorylation of CREB and HSP-27, which promote cell survival and inhibit apoptosis, was reduced by OLE [90,91].

We also found increased phosphorylation of c-jun and AKT upon OLE treatment. These results are difficult to explain, since the phosphorylation of c-jun and AKT usually promotes cell survival [92,93]. We may hypothesize that B-ALL cells reacted to OLE through the phosphorylation of these two factors in order to counterbalance the induction of apoptosis. Furthermore, the phosphorylation of PYK2, which mediated anti-apoptotic signaling of AKT, is decreased by OLE in B-ALL. Beyond the abovementioned relationship between CA-9, GSK3β, and AKT, it is to be taken into account that all these pathways underlying apoptosis and cell death are very complex, with an impressive number of molecules involved. Thus, it is conceivable that OLE triggers apoptosis as a result of a balance of several modulated molecules, which interact with each other. Here, we demonstrated for the first time to our knowledge that OLE, similarly to what we previously reported in neuroblastoma cells, acts as direct anti-tumor agent in B-ALL and with a lesser extent in AML cells, highlighting the potential molecular mechanisms involved, which are related to the induction of cellular stress and apoptosis, and are conceivably mediated by the extrinsic pathway, as witnessed by the highest modulation of TRAIL R1. Nonetheless, to unambiguously demonstrate such a conclusion, additional studies are required involving analysis of caspases and/or experiments focused on blocking death receptors such as TRAIL. The impact of OLE on these cellular pathways was modest in AML, resulting in a greater resistance to induction of apoptosis and inhibition of cell proliferation. Indeed, most of the proteins in AML were modulated in an opposite way compared to what was observed in B-ALL.

In summary, the effects of OLE on B-ALL cells are mainly related to (i) down-regulation of proteins involved in cancer progression, antioxidants, and heat-shock proteins, (ii) up-regulation of proteins related to apoptosis, and (iii) alteration of protein phosphorylation. The final effects based on these pathways are the inhibition of cell proliferation and induction of apoptosis. Thus, the resistance of AML cells to OLE is conceivably related to the minimal or absent alteration of these molecular pathways and, in addition, to a modulation of key proteins in the opposite way.

Interesting and promising data were obtained in experiments performed with OLE in combination with chemotherapeutic drugs. We selected two standard-of-care chemotherapeutic agents used for the management of acute leukemias and tested clinical-grade cytarabine and cyclophosphamide, as spare aliquots after therapeutic use in pediatric leukemic patients. In particular, cytarabine is currently used to treat patients with relapse/refractory disease [94,95], whereas cyclophosphamide represents a standard of care treatment for B-ALL patients [96]. Our data suggested that OLE may be used as a food supplement for these patients in combination with the drug to implement its anti-tumor efficacy. Notably, the presence or absence of synergistic and additive effects of OLE with cytarabine and cyclophosphamide, respectively, may conceivably be related to their different mechanisms of action. Indeed, cyclophosphamide acts as an alkylating agent, interfering with DNA replication and transcription of mRNA [97]. In contrast, cytarabine induces DNA damage through the incorporation of similar pyrimidine and purine nucleosides, leading to the activation of two kinase pathways, namely ataxia telangiectasia mutated and Rad3-related Checkpoint kinase 1 (ATR-CHK1) and ataxia telangiectasia mutated Checkpoint kinase 2 (ATM-CHK2), which may lead to DNA repair or apoptosis [98,99]. Due to the finding that OLE upregulated CHK2 in ALL (but not in AML) cell lines, we may speculate that the synergistic/additive effects observed with cytarabine may be related to the concomitant induction of CHK2 in target cells.

Pharmacokinetics and/or bioavailability of the different compounds present in OLE must be taken into account in view of clinical perspectives. Previous studies have addressed this issue, demonstrating that oleuropein and hydroxytyrosol are stable after digestion and reach their peak in the plasma of subjects consuming OLE within 20–75 min, and are still present 2–3 h after ingestion [100,101].

## 5. Conclusions

We here showed that exclusively the combination of OLE with cytarabine led to induction of apoptosis with effects ranging from synergy to addiction, whereas no synergic nor additive effects were observed when OLE was tested in combination with cyclophosphamide. These findings suggest that OLE not only has autonomous anti-tumor potential but can also increase the effectiveness of existing treatments in vitro. Although these data represent a proof-of-concept, we believe that a deep mechanistic insight aimed at validating the data raised from this study should be addressed in the future. Furthermore, our results highlighted a potential use of OLE in the clinical setting and management of pediatric B-ALL patients. Notably, recent technologies delineate areas for new interactions between food engineering and the medical field to aid in the best efficacy of cancer drugs, and to ensure more attractive and proper nutrition for cancer patients. In this view, it is to be taken into mind that OLE is an aqueous nutraceutical that may be easily administered as a food integrator without any side effects. Thus, we may envisage clinical settings with the use of OLE as an adjuvant compound during chemotherapy with cytarabine in pediatric oncology, taking advantage of its anti-proliferative and pro-apoptotic effects on B-ALL cells described here and of the already known anti-inflammatory properties. However, this hypothesis needs to be validated in future clinical studies to confirm the potential effectiveness of OLE as a food supplement in combination with existing therapies for B-ALL patients.

## Figures and Tables

**Table 1:** Characteristics of patients. Leukemia blasts were obtained from patients (Pt) at the time of diagnosis, at onset, and, in cases, at relapse.

Patients	Disease	Age (Years)	Leukemia Blasts
Pt #1	B-ALL	17	diagnosis
			relapse
Pt #2	B-ALL	5	diagnosis
			relapse
Pt #3	B-ALL	9	diagnosis
			relapse
Pt #4	B-ALL	8	diagnosis
Pt #5	B-ALL	4	diagnosis
Pt #6	AML	12	diagnosis
Pt #7	AML	10	diagnosis
Pt #8	AML	2	diagnosis

## Data Availability

The original contributions presented in this study are included in the article/Supplementary Materials. Further inquiries can be directed to the corresponding author.

## Supplementary Materials

The following supporting information can be downloaded at: https://www.mdpi.com/article/10.3390/nu18010015/s1, Figure S1: OLE displays synergistic effects with cytarabine; Figure S2: OLE did not show any synergy or additive effects with cyclophosphamide; Figure S3: Heat maps of OLE/cytarabine in NALM-6 cells; Figure S4: Heat maps of OLE/cyclophosphamide synergistic effects in NALM-6 cells; Figure S5: Heat maps of OLE/cytarabine synergistic effects in 697 cells; Figure S6: Heat maps of OLE/cyclophosphamide synergistic effects in 697 cells.

## Abbreviations

The following abbreviations are used in this manuscript:

OLE	Olive leaf extract
B-ALL	B-acute lymphoblastic leukemias
ROS	Reactive oxygen species
AML	Acute myeloid leukemias
CFSE	Carboxyfluorescein diacetate N-succinimidyl ester
