# Supplementary material for: Olive Leaf Extract (OLE) Anti-Tumor Activities Against Hematologic Tumors: Potential Therapeutic Implications for Pediatric Patients with B-Acute Lymphoblastic Leukemia
Source: Nutrients. 2025 Dec 19;18(1):15. doi: 10.3390/nu18010015 (PMC12787448; doi:10.3390/nu18010015)
Supplement: Supplementary file 1 [file nutrients-18-00015-s001.zip › nutrients-4014403-supplementary.pdf]

Figure S1

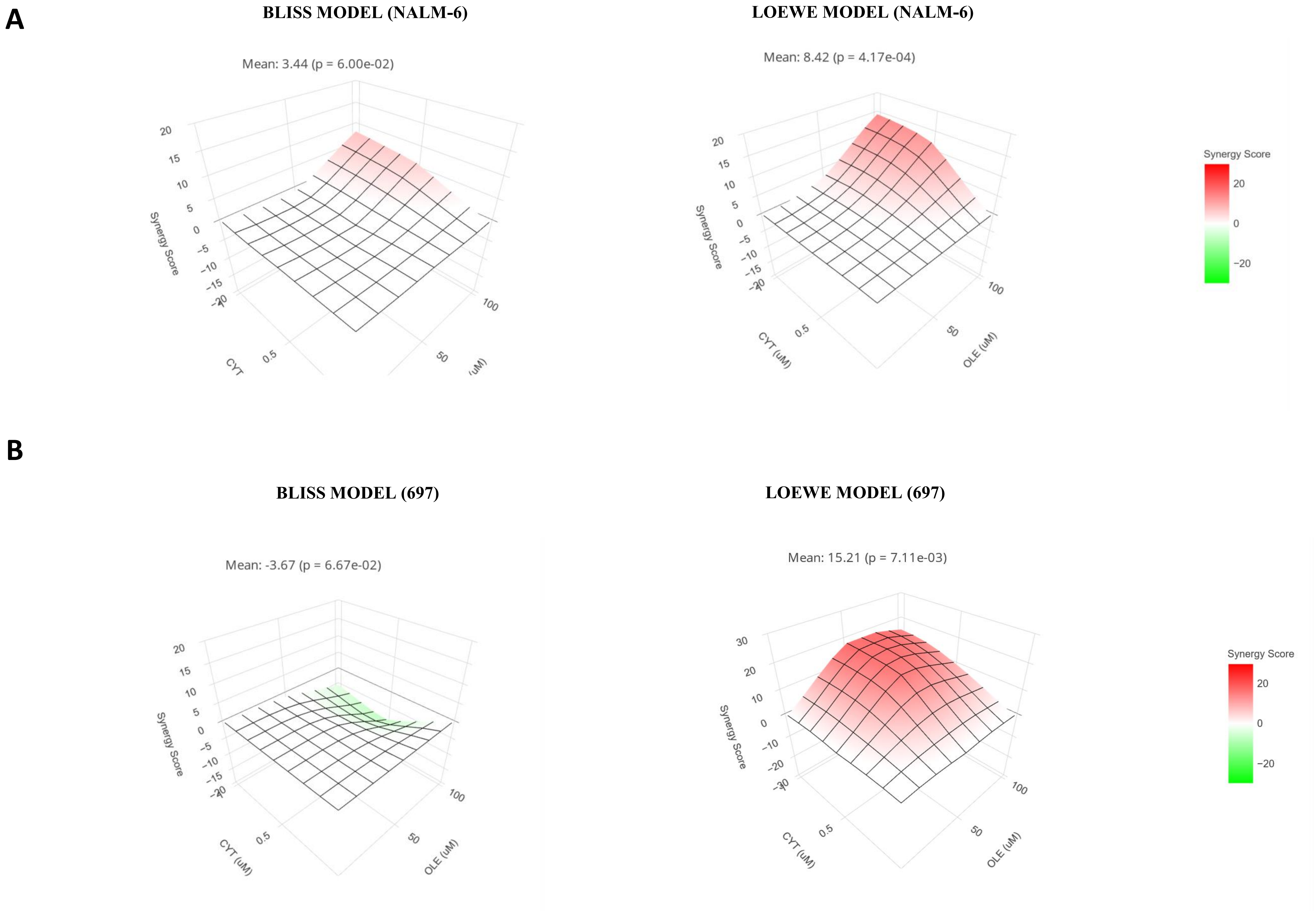
A
BLISS MODEL (NALM-6)
Mean: 3.44 (p = 6.00e-02)
Synergy Score
CYT
0.5
50
100
LOEWE MODEL (NALM-6)
Mean: 8.42 (p = 4.17e-04)
Synergy Score
CYT (uM)
0.5
OLE (uM)
50
100
Synergy Score
20
0
−20
B
BLISS MODEL (697)
Mean: -3.67 (p = 6.67e-02)
Synergy Score
CYT (uM)
0.5
OLE (uM)
50
100
LOEWE MODEL (697)
Mean: 15.21 (p = 7.11e-03)
Synergy Score
CYT (uM)
0.5
OLE (uM)
50
100
Synergy Score
20
0
−20

Figure S2

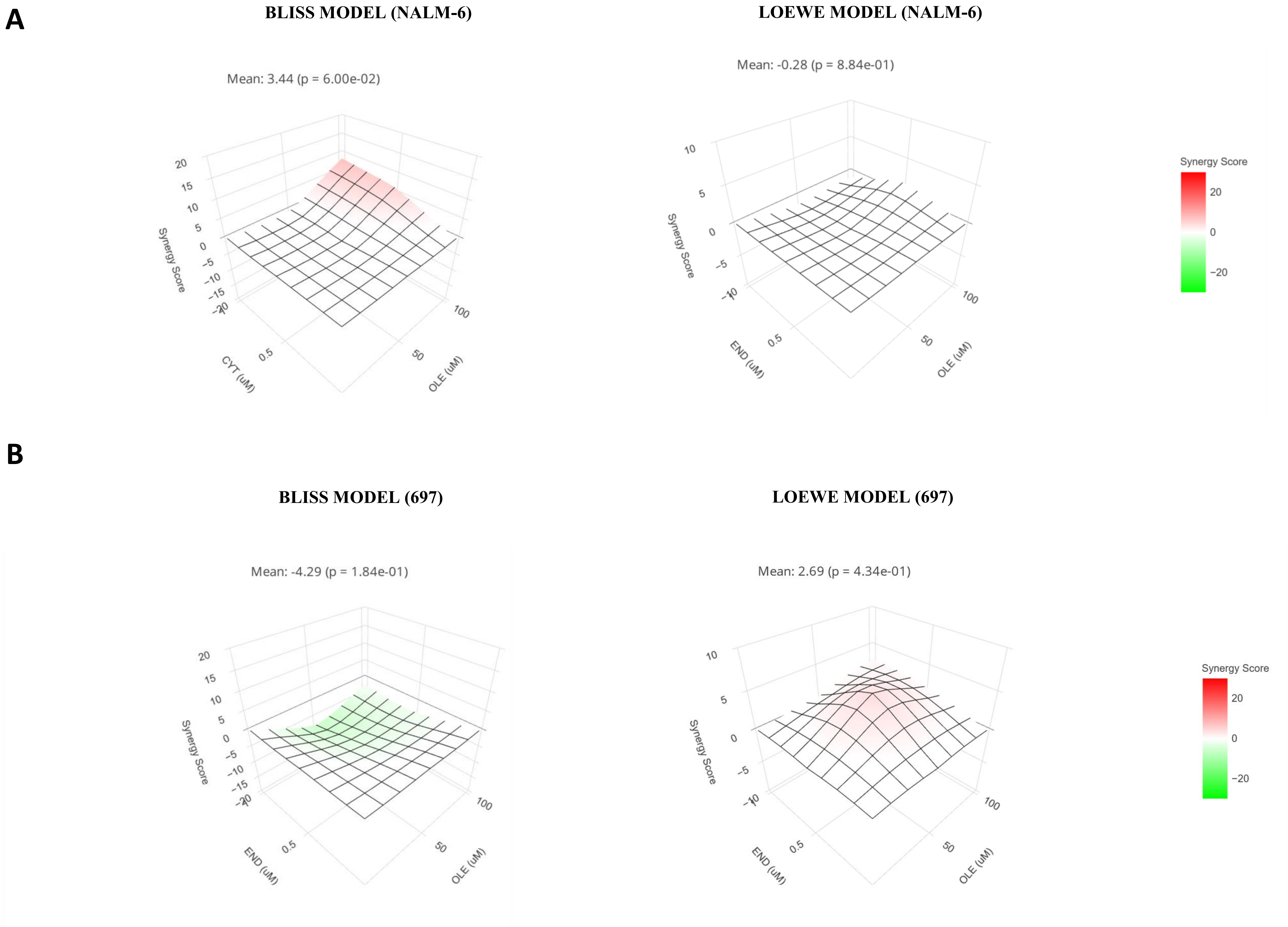
A
BLISS MODEL (NALM-6)
Mean: 3.44 (p = 6.00e-02)
Synergy Score
CYT (uM)
OLE (uM)
LOEWE MODEL (NALM-6)
Mean: -0.28 (p = 8.84e-01)
Synergy Score
END (uM)
OLE (uM)
Synergy Score
B
BLISS MODEL (697)
Mean: -4.29 (p = 1.84e-01)
Synergy Score
END (uM)
OLE (uM)
LOEWE MODEL (697)
Mean: 2.69 (p = 4.34e-01)
Synergy Score
END (uM)
OLE (uM)
Synergy Score

Figure S3

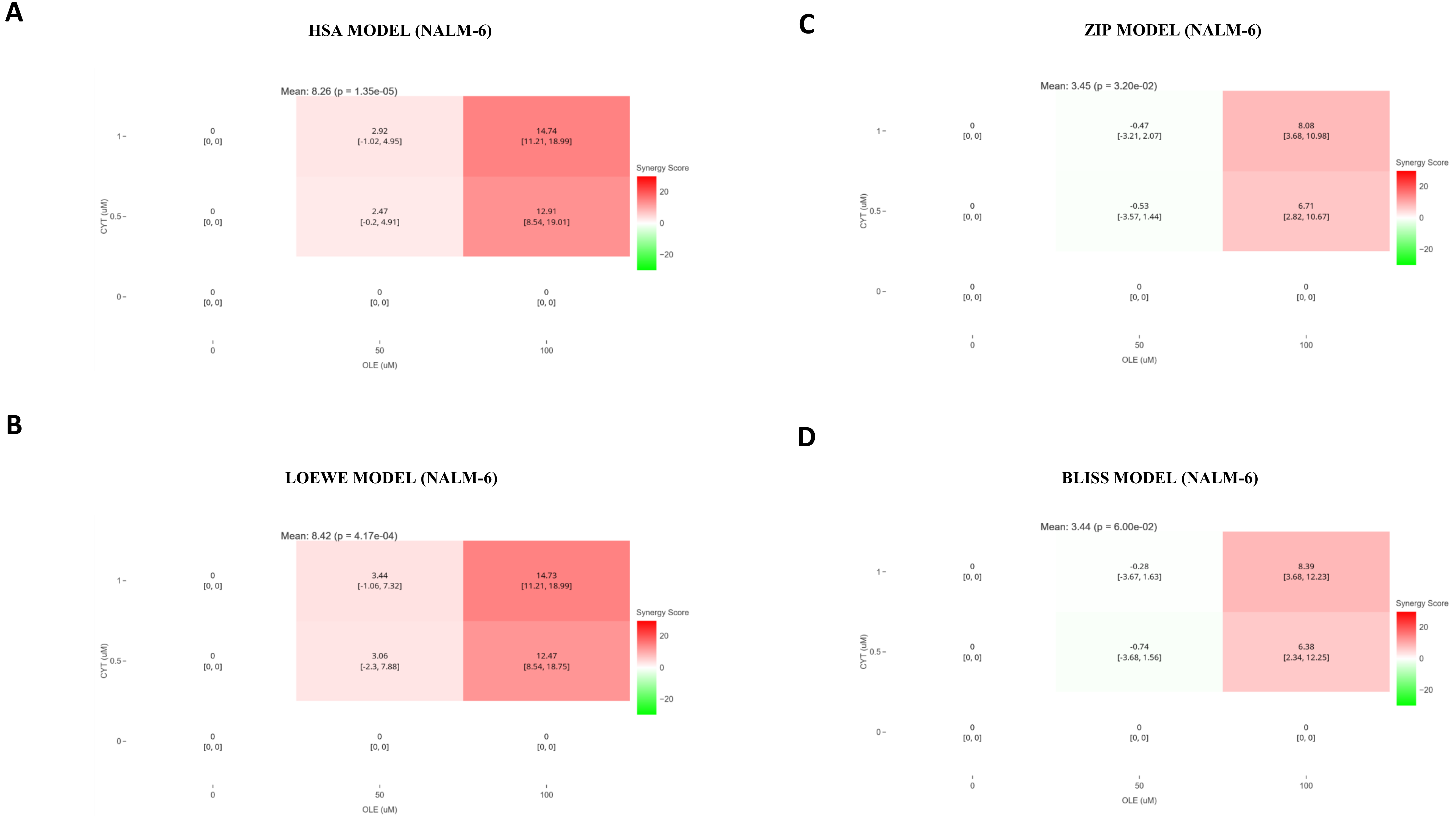
A
HSA MODEL (NALM-6)
Mean: 8.26 (p = 1.35e-05)
0 [0, 0]
2.92 [-1.02, 4.95]
14.74 [11.21, 18.99]
0 [0, 0]
2.47 [-0.2, 4.91]
12.91 [8.54, 19.01]
0 [0, 0]
0 [0, 0]
0 [0, 0]
CYT (uM)
OLE (uM)
Synergy Score
C
ZIP MODEL (NALM-6)
Mean: 3.45 (p = 3.20e-02)
0 [0, 0]
-0.47 [-3.21, 2.07]
8.08 [3.68, 10.98]
0 [0, 0]
-0.53 [-3.57, 1.44]
6.71 [2.82, 10.67]
0 [0, 0]
0 [0, 0]
0 [0, 0]
CYT (uM)
OLE (uM)
Synergy Score
B
LOEWE MODEL (NALM-6)
Mean: 8.42 (p = 4.17e-04)
0 [0, 0]
3.44 [-1.06, 7.32]
14.73 [11.21, 18.99]
0 [0, 0]
3.06 [-2.3, 7.88]
12.47 [8.54, 18.75]
0 [0, 0]
0 [0, 0]
0 [0, 0]
CYT (uM)
OLE (uM)
Synergy Score
D
BLISS MODEL (NALM-6)
Mean: 3.44 (p = 6.00e-02)
0 [0, 0]
-0.28 [-3.67, 1.63]
8.39 [3.68, 12.23]
0 [0, 0]
-0.74 [-3.68, 1.56]
6.38 [2.34, 12.25]
0 [0, 0]
0 [0, 0]
0 [0, 0]
CYT (uM)
OLE (uM)
Synergy Score

Figure S4

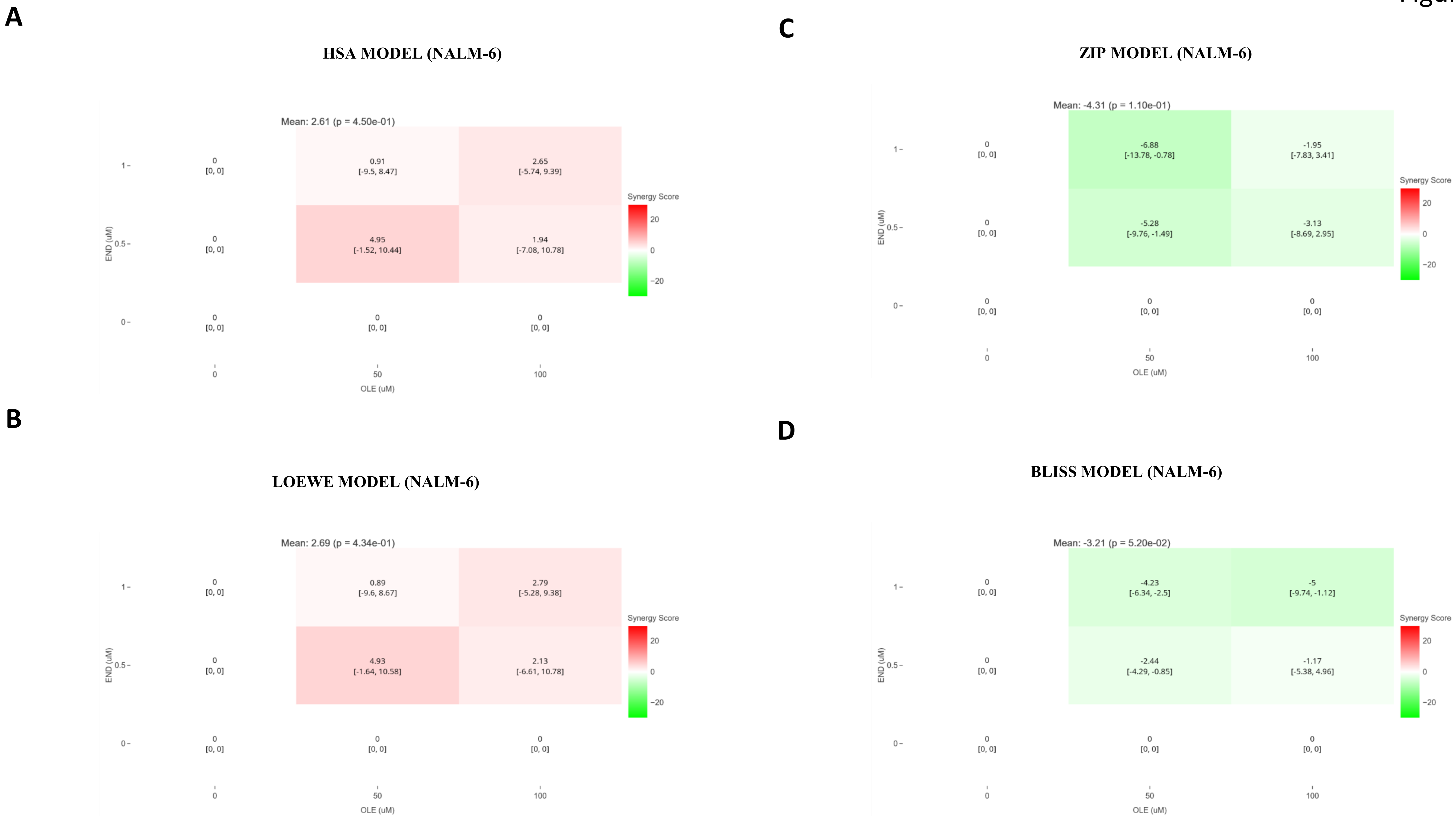
A
HSA MODEL (NALM-6)
Mean: 2.61 (p = 4.50e-01)
0 [0, 0]
0.91 [-9.5, 8.47]
2.65 [-5.74, 9.39]
0 [0, 0]
4.95 [-1.52, 10.44]
1.94 [-7.08, 10.78]
0 [0, 0]
0 [0, 0]
0 [0, 0]
END (uM)
1
0.5
0
OLE (uM)
0
50
100
Synergy Score
20
0
−20
B
LOEWE MODEL (NALM-6)
Mean: 2.69 (p = 4.34e-01)
0 [0, 0]
0.89 [-9.6, 8.67]
2.79 [-5.28, 9.38]
0 [0, 0]
4.93 [-1.64, 10.58]
2.13 [-6.61, 10.78]
0 [0, 0]
0 [0, 0]
0 [0, 0]
END (uM)
OLE (uM)
Synergy Score
C
ZIP MODEL (NALM-6)
Mean: -4.31 (p = 1.10e-01)
0 [0, 0]
-6.88 [-13.78, -0.78]
-1.95 [-7.83, 3.41]
0 [0, 0]
-5.28 [-9.76, -1.49]
-3.13 [-8.69, 2.95]
0 [0, 0]
0 [0, 0]
0 [0, 0]
END (uM)
OLE (uM)
Synergy Score
D
BLISS MODEL (NALM-6)
Mean: -3.21 (p = 5.20e-02)
0 [0, 0]
-4.23 [-6.34, -2.5]
-5 [-9.74, -1.12]
0 [0, 0]
-2.44 [-4.29, -0.85]
-1.17 [-5.38, 4.96]
0 [0, 0]
0 [0, 0]
0 [0, 0]
END (uM)
OLE (uM)
Synergy Score

Figure S5

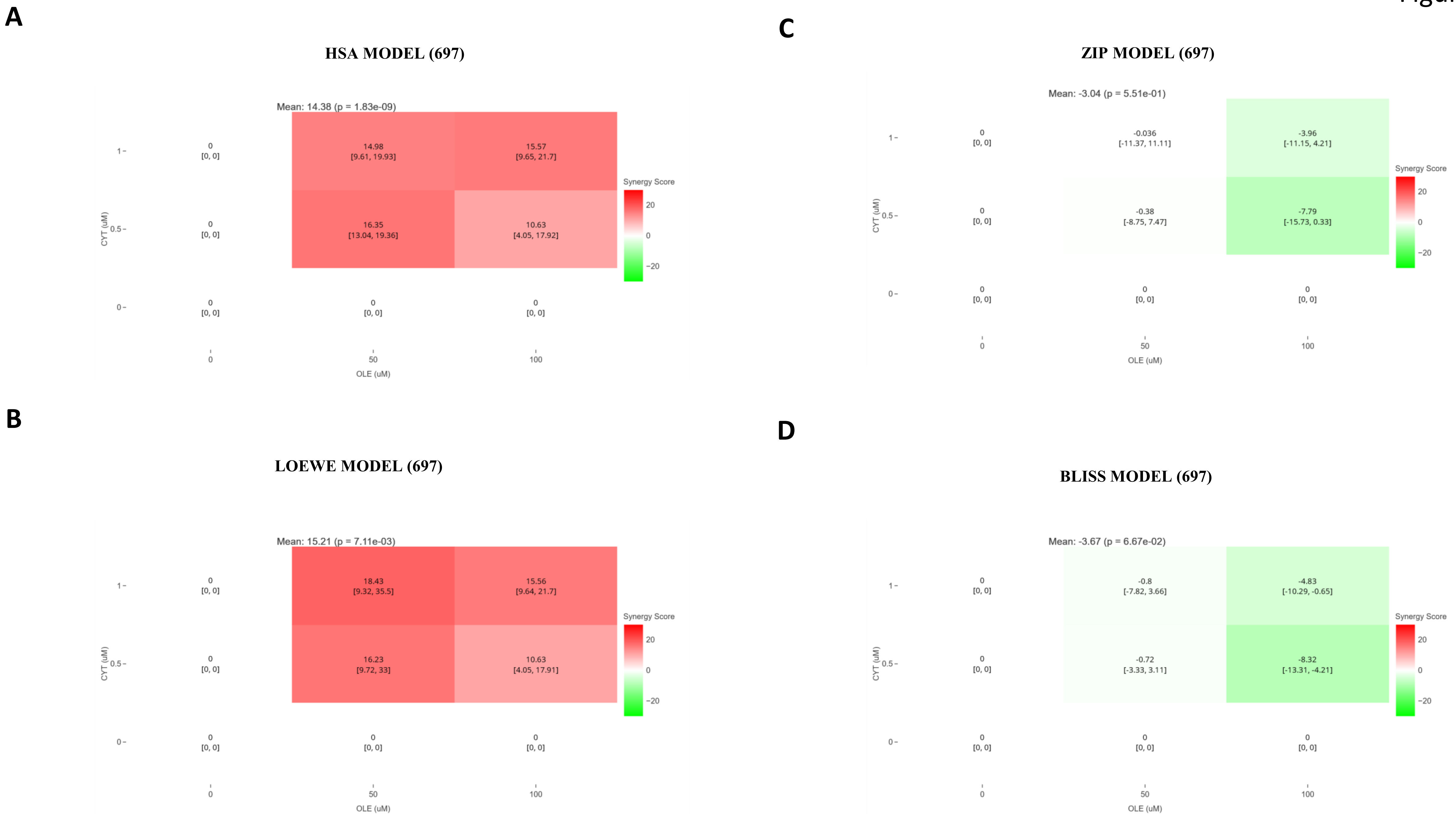
A
HSA MODEL (697)
Mean: 14.38 (p = 1.83e-09)
14.98 [9.61, 19.93]
15.57 [9.65, 21.7]
16.35 [13.04, 19.36]
10.63 [4.05, 17.92]
0 [0, 0]
Synergy Score
CYT (uM)
OLE (uM)
B
LOEWE MODEL (697)
Mean: 15.21 (p = 7.11e-03)
18.43 [9.32, 35.5]
15.56 [9.64, 21.7]
16.23 [9.72, 33]
10.63 [4.05, 17.91]
C
ZIP MODEL (697)
Mean: -3.04 (p = 5.51e-01)
-0.036 [-11.37, 11.11]
-3.96 [-11.15, 4.21]
-0.38 [-8.75, 7.47]
-7.79 [-15.73, 0.33]
D
BLISS MODEL (697)
Mean: -3.67 (p = 6.67e-02)
-0.8 [-7.82, 3.66]
-4.83 [-10.29, -0.65]
-0.72 [-3.33, 3.11]
-8.32 [-13.31, -4.21]

Figure S6

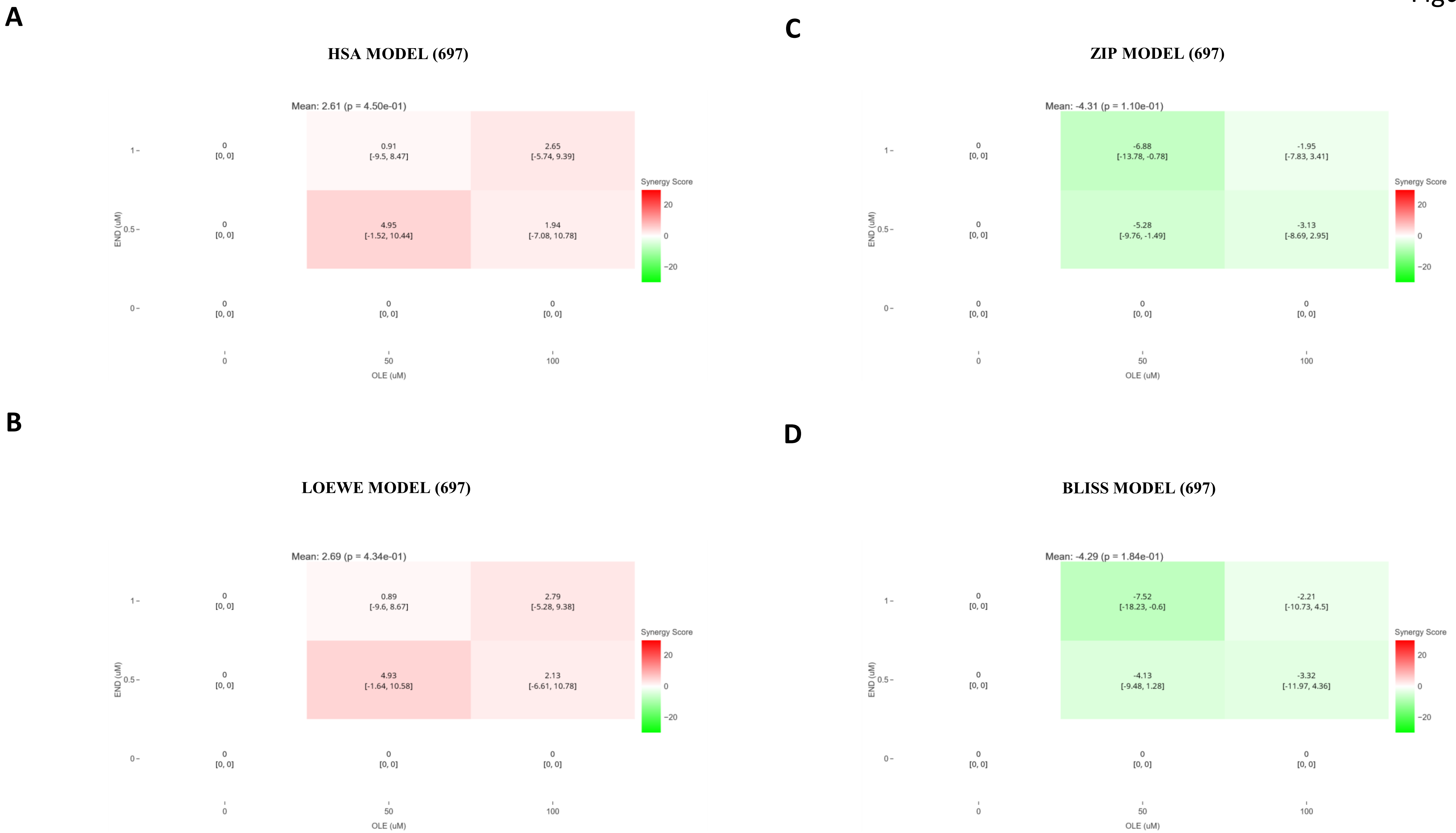
A
HSA MODEL (697)
Mean: 2.61 (p = 4.50e-01)
0 [0, 0]
0.91 [-9.5, 8.47]
2.65 [-5.74, 9.39]
0 [0, 0]
4.95 [-1.52, 10.44]
1.94 [-7.08, 10.78]
0 [0, 0]
0 [0, 0]
0 [0, 0]
END (uM)
1
0.5
0
OLE (uM)
0
50
100
Synergy Score
20
0
−20
B
LOEWE MODEL (697)
Mean: 2.69 (p = 4.34e-01)
0 [0, 0]
0.89 [-9.6, 8.67]
2.79 [-5.28, 9.38]
0 [0, 0]
4.93 [-1.64, 10.58]
2.13 [-6.61, 10.78]
0 [0, 0]
0 [0, 0]
0 [0, 0]
C
ZIP MODEL (697)
Mean: -4.31 (p = 1.10e-01)
0 [0, 0]
-6.88 [-13.78, -0.78]
-1.95 [-7.83, 3.41]
0 [0, 0]
-5.28 [-9.76, -1.49]
-3.13 [-8.69, 2.95]
0 [0, 0]
0 [0, 0]
0 [0, 0]
D
BLISS MODEL (697)
Mean: -4.29 (p = 1.84e-01)
0 [0, 0]
-7.52 [-18.23, -0.6]
-2.21 [-10.73, 4.5]
0 [0, 0]
-4.13 [-9.48, 1.28]
-3.32 [-11.97, 4.36]
0 [0, 0]
0 [0, 0]
0 [0, 0]

SUPPLEMENTAL FIGURES CAPTIONS

Figure S1. OLE displays synergistic effects with cytarabine. NALM-6 (**panel A)** and 697 (**panel B)** cell lines were cultured 48 h in the presence or absence of OLE (50 and 100 μM) and cytarabine (cyt, 0,5 and 1 μM) and the percentage of alive cells was evaluated for each experimental combination. Additive effects were analyzed by Bliss model (left panels) and synergistic effects by the Loewe model (right panels) Means and p values are shown.

Figure S2. OLE did not show any synergy or additive effects with cyclophosphamide. NALM-6 (**panel A)** and 697 (**panel B)** cell lines were cultured 48 h in the presence or absence of OLE (50 and 100 μM) and cyclophosphamide/endoxan (end 0,5 and 1 μg/ml) and the percentage of alive cells was evaluated for each experimental combination. Additive effects were analyzed by Bliss model (left panels) and synergistic effects by the Loewe model (right panels) Means and p values are shown.

Figure S3. Heat maps of OLE/cytarabine in NALM-6 cells. The NALM-6 cell line was cultured 48 h in the presence or absence of OLE (50 and 100 μM) and cytarabine (cyt 0,5 and 1 μM) and the percentage of alive cells was evaluated for each experimental combination. Synergistic effects were analyzed by HSA (panel A) and Loewe (panel B) models, whereas additive effects by ZIP (panel C) and Bliss (panel D) models. Means and p values are shown.

Figure S4. Heat maps of OLE/cyclophosphamide synergistic effects in NALM-6 cells. The NALM-6 cell line was cultured with or without OLE (50 and 100 μM) and cyclophosphamide/endoxan (end 0,5 and 1 μM) and the percentage of alive cells was evaluated for each experimental combination. Synergistic effects were analyzed by HSA (panel A) and Loewe (panel B) models, whereas additive effects by ZIP (panel C) and Bliss (panel D) models. Means and p values are shown.

Figure S5. Heat maps of OLE/cytarabine synergistic effects in 697 cells. The 697 cell line was cultured 48 h in the presence or absence of OLE (50 and 100 μM) and cytarabine (cyt 0,5 and 1 μM)

and the percentage of alive cells was evaluated for each experimental combination. Synergistic effects were analyzed by HSA (panel A) and Loewe (panel B) models, whereas additive effects by ZIP (panel C) and Bliss (panel D) models. Means and p valued are shown.

Figure S6. Heat maps of OLE/cyclophosphamide synergistic effects in 697 cells. The 697 cell line was cultured 48 h in the presence or absence of OLE (50 and 100 μM) and cyclophosphamide/endoxan (end 0,5 and 1 μM) and the percentage of alive cells was evaluated for each experimental combination. Synergistic effects were analyzed by HSA (panel A) and Loewe (panel B) models, whereas additive effects by ZIP (panel C) and Bliss (panel D) models. Means and p valued are shown.
